# SARS-CoV-2 Spike Protein 1 Activates Microvascular Endothelial Cells and Complement System Leading to Platelet Aggregation

**DOI:** 10.3389/fimmu.2022.827146

**Published:** 2022-03-07

**Authors:** Luca Perico, Marina Morigi, Miriam Galbusera, Anna Pezzotta, Sara Gastoldi, Barbara Imberti, Annalisa Perna, Piero Ruggenenti, Roberta Donadelli, Ariela Benigni, Giuseppe Remuzzi

**Affiliations:** ^1^ Istituto di Ricerche Farmacologiche Mario Negri IRCCS, Bergamo, Italy; ^2^ Unit of Nephrology and Dialysis, Azienda Socio Sanitaria Territoriale (ASST) Papa Giovanni XXIII, Bergamo, Italy

**Keywords:** COVID-19, SARS-CoV-2 spike protein 1, complement system, endothelial dysfunction, inflammation, thrombosis

## Abstract

Microvascular thrombosis is associated with multiorgan failure and mortality in coronavirus disease 2019 (COVID-19). Although thrombotic complications may be ascribed to the ability of SARS-CoV-2 to infect and replicate in endothelial cells, it has been poorly investigated whether, in the complexity of viral infection in the human host, specific viral elements alone can induce endothelial damage. Detection of circulating spike protein in the sera of severe COVID-19 patients was evaluated by ELISA. *In vitro* experiments were performed on human microvascular endothelial cells from the derma and lung exposed to SARS-CoV-2-derived spike protein 1 (S1). The expression of adhesive molecules was studied by immunofluorescence and leukocyte adhesion and platelet aggregation were assessed under flow conditions. Angiotensin converting enzyme 2 (ACE2) and AMPK expression were investigated by Western Blot analysis. In addition, S1-treated endothelial cells were incubated with anti-ACE2 blocking antibody, AMPK agonist, or complement inhibitors. Our results show that significant levels of spike protein were found in the 30.4% of severe COVID-19 patients. *In vitro*, the activation of endothelial cells with S1 protein, *via* ACE2, impaired AMPK signalling, leading to robust leukocyte recruitment due to increased adhesive molecule expression and thrombomodulin loss. This S1-induced pro-inflammatory phenotype led to exuberant C3 and C5b-9 deposition on endothelial cells, along with C3a and C5a generation that further amplified S1-induced complement activation. Functional blockade of ACE2 or complement inhibition halted S1-induced platelet aggregates by limiting von Willebrand factor and P-selectin exocytosis and expression on endothelial cells. Overall, we demonstrate that SARS-CoV-2-derived S1 is sufficient in itself to propagate inflammatory and thrombogenic processes in the microvasculature, amplified by the complement system, recapitulating the thromboembolic complications of COVID-19.

## Introduction

In December 2019, a novel coronavirus was isolated from the respiratory epithelium of patients with unexplained pneumonia in Wuhan, China. This pathogen, named Severe Acute Respiratory Syndrome Coronavirus 2 (SARS-CoV-2), was identified as the causative agent of Coronavirus Disease 2019 (COVID-19). As of January 2022, over 375 million confirmed SARS-CoV-2 cases had been reported, claiming almost 5.7 million lives worldwide (1).

SARS-CoV-2 is a highly cytopathic virus that, like the other members of the *coronaviridae* family, induces epithelial cells to undergo apoptotic cell death as part of its replication cycle (2). Following SARS-CoV-2 infection in target cells (3, 4), the disease can manifest as a series of different clinical conditions, ranging from asymptomatic to life-threatening cases (5). For about 80% of patients, the infection is restricted to the proximal airways of the lungs, causing mild disease with modest symptoms (5). In about 20% of patients, SARS-CoV-2 infection can expand to the distal lung and rapidly deteriorate to a severe illness, characterised by bilateral interstitial pneumonia, acute respiratory distress syndrome (ARDS), and multi-organ damage with a high fatality rate (5).

Among the distinctive features of severe COVID-19, vascular abnormalities have been among the most frequently reported complications (6–8). There is growing evidence that SARS-CoV-2 induces endotheliitis and inflammatory cell infiltration in the lungs (7, 9). Following endothelial infection, loss of vessel barrier integrity and the development of a pro-coagulative endothelium have been identified as pivotal contributors to the initiation and propagation of ARDS (7, 9). Evidence of altered coagulation parameters during COVID-19 appeared in early reports from China, as revealed by elevated levels of partial thromboplastin time, prothrombin, D-dimer, and C-reactive protein in hospitalized patients (10). Similarly, autoptic studies in lung tissues found the presence of platelet-fibrin thrombi in small vessels associated with foci of alveolar hemorrhage (11, 12), suggesting that coagulopathy is critical for the outcome of COVID-19 (13–15). In this context, microvascular injury and thrombosis have also been shown to be associated with the activation of the complement system, as revealed by the presence of C3, C3a, C5a, and C5b-9 in the lungs and in the circulation of patients who succumbed to SARS-CoV-2 infection (16–19). In line with these findings, overt disseminated intravascular coagulation of small and large vessels (20), complement activation, and venous thromboembolic complications, in particular acute pulmonary embolisms (21–24), were identified as the pathogenic features of non-survivor COVID-19 patients.

Mechanistically, several data pointed to an immune system over-reaction as the main driver of the disruption of the thromboresistant phenotype of the microvascular endothelium during SARS-CoV-2 infection (25). Additional findings also revealed that SARS-CoV-2 can induce vascular damage in the lungs by directly infecting endothelial cells. This hypothesis has been corroborated by electron microscopy and immunofluorescence analyses of post-mortem tissue that showed that SARS-CoV-2 and viral particles can be detected in endothelial cells within the lungs (7, 9), although the clinical (26–28) and experimental data (29, 30) here are controversial. Spatially resolved SARS-CoV-2 RNA was detected in the pulmonary endothelium through *in situ* hybridization (31), further suggesting potential viral replication in lung microvascular endothelial cells. Although with mixed results (32, 33), a recent study suggested that SARS-CoV-2 can be found in the circulation more abundantly than previously thought and that plasmatic viremia correlates with disease severity and mortality (34). Based on this finding, it is conceivable that endothelial cells are exposed to SARS-CoV-2 in severe COVID-19 patients.

Of all the SARS-CoV-2 components, the subunit 1 of the spike protein (S1) – which is generated following proteolysis by host proteases such as TMPRSS2 (35, 36) – has the ability to interact with different receptors on the human target cells, including angiotensin converting enzyme 2 (ACE2), *via* the receptor-binding domain (RBD) and can induce specific cellular responses (35, 36). Numerous studies have demonstrated that endothelial cells express both ACE2 and TMPRSS2 (37–39), suggesting that the S1 protein potentially has an effect on endothelial cell activation and dysfunction, possibly leading to the engagement of pro-apoptotic pathways (40). In relation to this, the deposition of S1 protein has been documented in the cutaneous microvascular endothelium (41). In line with the above findings, it has also been shown that SARS-CoV-2-derived S1 protein, but not other structural proteins of the virus, can bind endothelial cells, inducing alterations in endothelial cell phenotype by enhancing the expression of cytokines, adhesive molecules and reactive oxygen species, as well as impairing cell permeability and metabolic functions (42–46). In addition, it has been shown that S1 can directly activate the alternative pathway of complement on the cell surface by interfering with Factor H function (47). Lastly, our group recently documented that exposing sera from severe COVID-19 patients to endothelial cells induced platelet aggregation *via* the engagement of C5a/C5aR1 axis (48).

The aim of this study is to investigate whether SARS-CoV-2-derived S1 is in itself sufficient to alter the endothelial phenotype, leading to microvascular inflammatory response and thrombosis *via* activation of the complement system.

## Results

### SARS-CoV-2-Derived Spike Protein Is Detectable in the Circulation of Patients With Severe COVID-19

We explored the presence of the spike protein in sera from uninfected subjects (n=9), mildly ill convalescent COVID-19 patients (n=9) and severely ill COVID-19 patients (n=23) with an enzyme-linked immunosorbent assay (ELISA). Patients’ characteristics are summarized in **Table 1**.

**Table 1:** Baseline characteristics of patients included in the study.

	Overall (n=41)	Negative control (n=9)	Mild COVID-19 (n=9)	Severe COVID-19 (n=23)	*p*-value
Age (years)*	62.6 ± 14.5	58.9 ± 12.1^a,b^	59.0 ± 19.0^c^	65.5 ± 13.4	^a^0.210 *vs* Severe COVID-19 ^b^0.284 *vs* Mild COVID-19 ^c^0.988 *vs* Severe COVID-19
Male sex (%)	26 (63.4)	6 (66.7)^d,e^	5 (55.6)^f^	15 (65.2)	^d^0.938 *vs* Severe COVID-19 ^e^0.629 *vs* Mild COVID-19 ^f^0.612 *vs* Severe COVID-19

*mean ± S.D.

The superscript refers to individual p-value of each parameter.

None of the selected sera from uninfected subjects or mildly ill COVID-19 patients (0 out of 18; 0%) exhibited detectable levels of circulating spike protein. Conversely, we found that 30.4% (7 out of 23) of hospitalized COVID-19 patients with active disease had detectable levels of spike protein above the detection range of the assay. Individual levels of the spike protein in hospitalized patients’ sera are reported in **Table 2**.

**Table 2:** Levels of the spike protein in the sera of hospitalized COVID-19 patients.

Patient	Age(years)	Gender	Spike protein levels*(ng/ml)
1	79	Female	45.51
2	60	Male	16.72
3	77	Male	9.43
4	78	Male	3.07
5	61	Female	2.94
6	49	Male	2.76
7	27	Male	2.73
8	76	Male	Negative
9	55	Male	Negative
10	63	Male	Negative
11	71	Male	Negative
12	66	Male	Negative
13	44	Female	Negative
14	69	Female	Negative
14	76	Male	Negative
16	67	Female	Negative
17	78	Male	Negative
18	77	Male	Negative
19	80	Female	Negative
20	76	Male	Negative
21	71	Male	Negative
22	56	Female	Negative
23	60	Female	Negative

*Assay sensitivity: > 2.7 ng/ml.

When hospitalized COVID-19 patients were divided according to positivity for spike protein in the serum, we found that the mean age of spike protein-positive patients was similar to that of spike-negative patients (**Table 3**). There were no differences between the sexes regarding the rate of spike protein positivity (**Table 3**). In contrast, there was a borderline significant difference (*p-value*=0.057) in the rate of SARS-CoV-2 RNA positivity when we used RT-real-time PCR on nasopharyngeal samples at the time of blood withdrawal (**Table 3**), suggesting that spike protein-positive patients still had detectable viral load at the time of hospitalization. This was further supported by the finding – though it was not statistically significant – that spike-positive patients tended to be admitted early after symptom onset compared to spike-negative subjects (**Table 3**). Our data are in line with previous findings showing that viral peak occurs early, 2-4 days after infection, while viral shedding is almost absent after 10 days (32, 49–51).

**Table 3:** Baseline characteristics of severe COVID-19 patients divided according to serum positivity to spike protein.

	Overall (n=23)	Spike-negative (n=16)	Spike-positive (n=7)	*p*-value
Age (years)*	65.5 ± 13.4	67.2 ± 10.5	61.6 ± 19.0	0.368
Male sex (%)	15 (65.2)	10 (62.5)	5 (71.4)	0.146
Patients with positive RT-PCR at hospital admission (%)	16 (69.6)	9 (56.2)	7 (100)	0.057
Length from symptom onset to hospital admission (days)*	8.1 ± 4.1	9.1 ± 4.3	5.9 ± 2.6	0.084
Comorbidities				
Hypertension (%)	8 (47.8)	3 (50)	3 (42.9)	0.752
Cardiovascular (%)	6 (26.1)	4 (25)	2 (28.6)	0.857
Diabetes (%)	7 (30.4)	5 (31.3)	2 (28.6)	0.898
Obesity (%)	4 (17.4)	2 (12.5)	2 (28.5)	0.349
Clinical and biochemical parameters				
BMI (Kg/m^2^)*	26.5 ± 4.0	26.2 ± 4.3	27.1 ± 4.1	0.747
WBC (cells/mL)*	9315 ± 3264	9106 ± 3494	9870 ± 2756	0.637
PLTs (cells/mL)*	255826 ± 140194	264938 ± 130483	235000 ± 670248	0.575
CRP (mg/dL)*	12.2 ± 8.6	11.3 ± 8.0	14.6 ± 10.4	0.426
LDH (U/L)*	451 ± 308	348 ± 120	627 ± 449	0.054
D-dimer (ng/mL)*	3065 ± 8089	4013 ± 9442	600 ± 345	0.439
PF (mmHg)*	148 ± 47	147 ± 53	151 ± 26	0.271
Complement components				
C5a (ng/ml)*	41.4 ± 20.6	27.9 ± 13.0	60.4 ± 11.8	0.001
sC5b-9 (ng/ml)*	1137 ± 432	987 ± 292	1347 ± 539	0.164
Patients receiving antithrombotic treatments before admission (%)	7 (30.4)	3 (18.7)	4 (57.1)	0.066
Hospitalization length (days)*	41.8 ± 33.7	46.8 ± 32.4	30.4 ± 36.3	0.293
Deaths (%)	7 (30.4)	5 (31.3)	2 (28.6)	0.898

*mean ± S.D.

BMI, body mass index; CRP, c-reactive protein; LDH, lactate dehydrogenases; PF, PaO_2_/FiO_2_ ratio; PLTs, platelets; WBC, white blood cell.

No comorbidities were found to be associated with a positive result in the detection of the spike protein (**Table 3**). While we found no differences in most of the clinical and biochemical parameters analysed, we observed a borderline difference (*p-value*=0.054) in the levels of lactate dehydrogenase (LDH) found in spike-positive and -negative patients (**Table 3**), possibly reflecting increased, widespread tissue damage in spike-positive patients. When we measured circulating levels of C5a and sC5b-9, we found that COVID-19 patients had significantly increased levels of C5a and sC5b-9 compared to healthy subjects (C5a: 41.4 ± 20.6 vs 7.6 ± 2.2 ng/ml, *p-value*<0.0001; sC5b9: 1137 ± 432 vs 205 ± 59 ng/ml, *p-value*<0.0001). In spike-positive patients, we found significantly higher levels of circulating C5a compared to spike-negative patients (C5a: 60.4 ± 11.8 vs 27.9 ± 13.0 ng/ml; *p-value*=0.0013, **Table 3**).

We were not able to observe a statistical difference in the % of subjects experiencing thrombotic events between spike-positive and -negative patients when we analysed their medical records, although all thrombotic complications occurred in spike-negative subjects (**Table 3**). The explanation for this unexpected finding is that most spike-positive COVID-19 patients, unlike those who were spike-negative, were receiving anticoagulant treatments before hospital admission (**Table 3**), possibly counteracting the thrombogenic effect of SARS-CoV-2.

In line with a previous finding in a similar geographical area during the same time period (52), here we also found that thromboembolic complications, which occurred in 26.1% of severe COVID-19 cases, manifested at hospital admission, possibly suggesting that thrombosis is a hallmark feature of advanced disease. As for the outcome, we found no differences between the two groups regarding hospitalization length and death (**Table 3**).

### SARS-CoV-2-Derived S1 Protein Binds Endothelial Cells and Alters Their Phenotype in a Dose-Dependent Manner

Having identified detectable levels of SARS-CoV-2 spike protein in the circulation of severe COVID-19 patients, we evaluated whether spike protein-derived S1 was in itself sufficient to alter endothelial cell phenotype.

**Figure 1:**
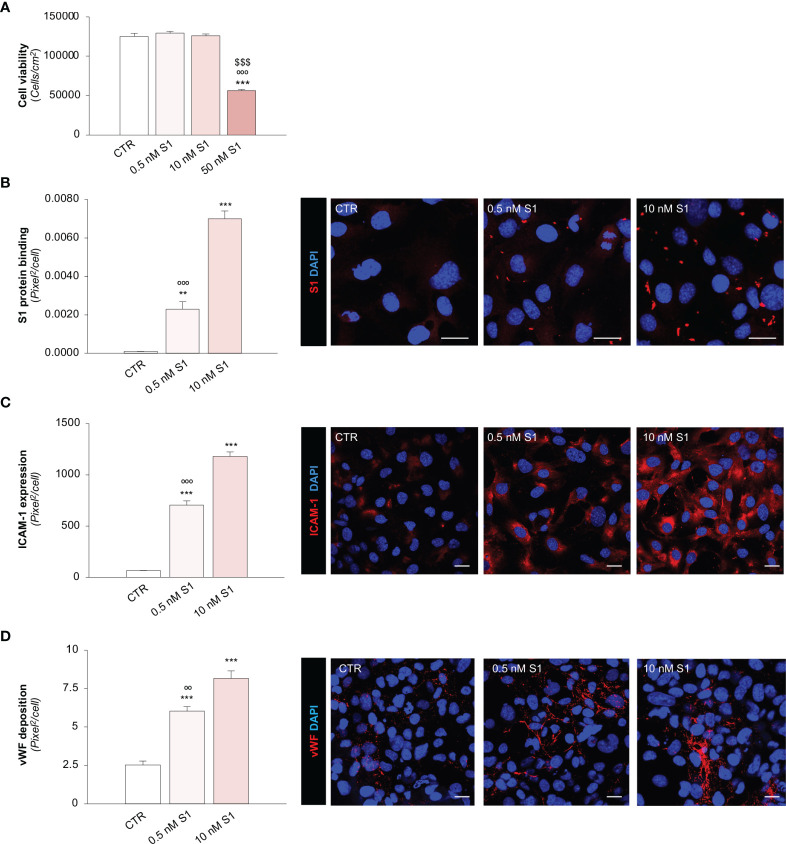
SARS-CoV-2-derived Spike 1 (S1) protein affects microvascular endothelial viability and phenotype *in vitro*. **(A)** Quantification of cell viability in HMEC-1 exposed for 24 h to medium alone (CTR) or S1 at the concentration of 0.5 nM, 10 nM, or 50 nM. **(B)** Quantification and representative images of the binding of the S1 protein (red) to HMEC-1 treated with medium alone (CTR) or S1 at the concentration of 0.5 nM and 10 nM for 24 h. **(C, D)** Quantification and representative images of ICAM-1 expression [**(C)**, red] and vWF deposition [**(D)**, red] on HMEC-1 treated with medium alone (CTR) or S1 at the concentration of 0.5 nM and 10 nM for 24 h. All experiments were repeated 3 times. Data represent mean ± SEM and were analysed with Tukey’s multiple comparison test. **p-value<0.01, and ***p-value<0.001 *vs* CTR; ^$$$^ *p-value<*0.001 *vs* 0.5 nM S1; °°*p-value<*0.01, and °°°*p-value<*0.001 *vs* 10 nM S1. All the slides were counterstained with DAPI (blue). Scale bar 20 μm.

First, we evaluated the effects of different S1 concentrations (53, 54) on human microvascular endothelial cell (HMEC-1) viability, which went from a concentration similar to that found in the sera of severe COVID-19 patients to higher concentrations used in previous studies (42, 47, 53, 54). As shown in **Figure 1A**, at the concentrations of 0.5 and 10 nM, S1 did not affect endothelial cell count after 24 h exposure, unlike when a concentration of 50 nM S1 was used, which markedly reduced cell vitality at 24 h. These findings were corroborated by data obtained with a crystal violet viability assay, showing a significant decrease in endothelial cell viability only after exposure to 50 nM S1 (**Supplementary Figure 1A**).

Having established the sub-toxic concentrations of S1, we elected to use 0.5 and 10 nM S1 in the subsequent experiments. First, we evaluated whether S1 can bind and activate endothelial cells. Through immunofluorescence analysis, we found a significant dose-dependent binding of the S1 protein on the apical surface of HMEC-1 treated with 0.5 and 10 nM S1 (**Figure 1B**). Then, we evaluated whether the binding of S1 could alter the phenotype of endothelial cells by analysing the expression of the pro-inflammatory adhesive molecule for leukocyte, intercellular adhesion molecule 1 (ICAM-1) (55), and the pro-thrombogenic protein von Willebrand factor (vWF) (56). We found that both S1 concentrations were able to significantly upregulate ICAM-1 protein expression (**Figure 1C**) in a dose-dependent manner and enhance vWF deposition (**Figure 1D**). Given the critical thrombogenic role of vWF when shuttled from Weibel-Palade to the endothelial luminal surface (56), we elected to analyse its localization through a 3D reconstruction of z-stack slices acquired with confocal microscopy. In resting HMEC-1 co-stained with green cell tracker, we found that vWF was detectable in the cell cytoplasm, in contrast with 10 nM S1-activated endothelial cells, which exhibited remarkable vWF staining on the luminal surface (**Supplementary Figure 1B**), reflecting the ability of S1 to promote the release of vWF a key player in the formation of platelet adhesion and aggregation.

Collectively, these findings highlight a novel mechanism triggered by SARS-CoV-2-derived S1 alone that can propagate the pro-inflammatory and pro-thrombotic effects on the endothelium.

Given that we found detectable levels of the spike protein in the sera of hospitalized patients, it is conceivable that the total burden of circulating S1 on endothelial cells could be significantly higher in the early course of the disease. SARS-CoV-2 viral load peacks early after symptom onset, while it decreases at 10 days, when patients are usually hospitalized (32, 49–51). For this reason, we used the sub-toxic concentration of 10 nM S1 in subsequent experiments to mimic the prolonged exposure of endothelial cells to S1 that occurs during the early phase of COVID-19.

### SARS-CoV-2-Derived S1 Protein Induces Pro-Inflammatory and Pro-Thrombotic Responses in Endothelial Cells Through the Engagement of ACE2

**Figure 2:**
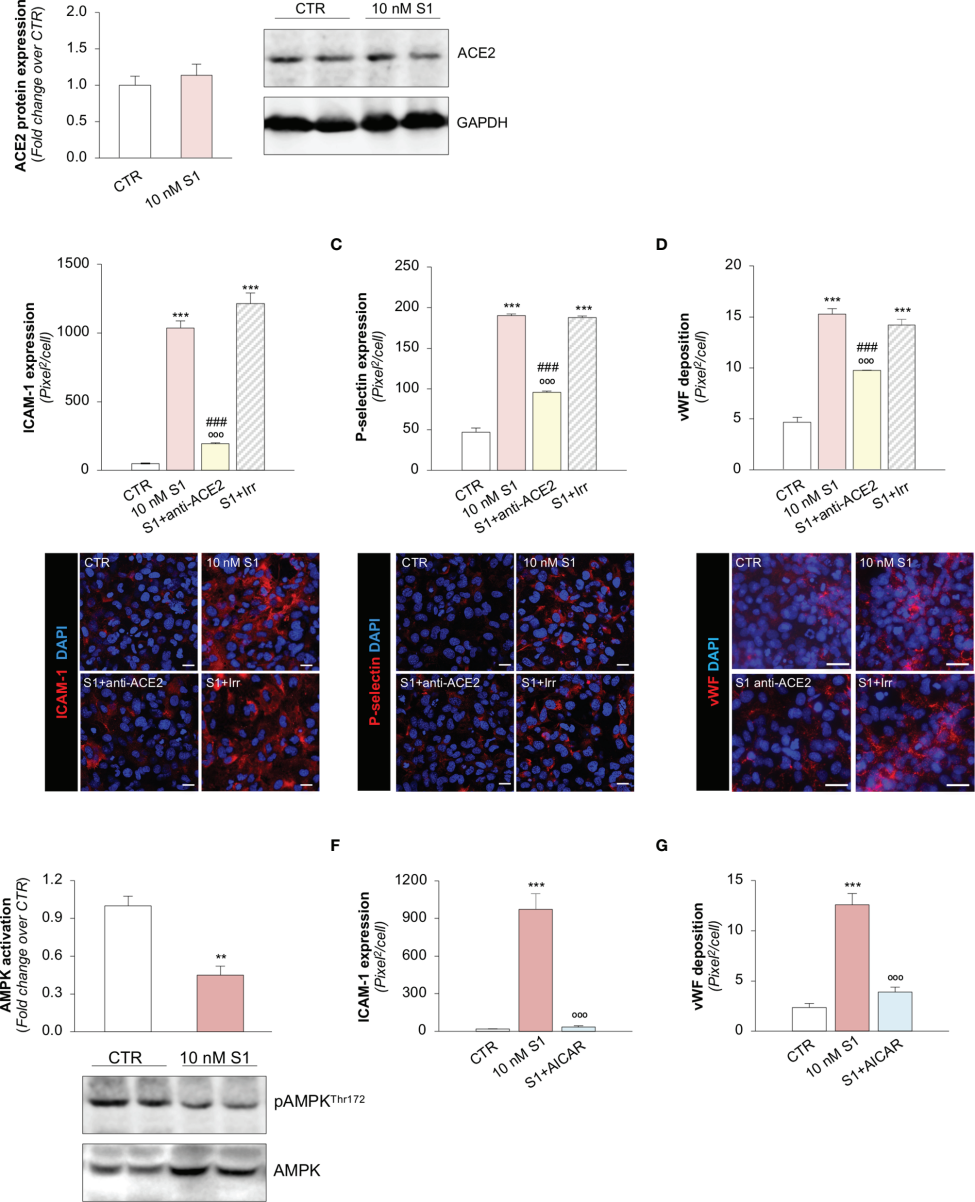
S1, through ACE2, upregulates adhesive molecules on HMEC-1 by impairing AMPK signalling. **(A)** Quantification and representative Western Blots of ACE2 protein expression in HMEC-1 exposed for 24h to medium alone (CTR) or S1 (10 nM). GAPDH was used as a sample loading control. **(B–D)** Quantification and representative images of ICAM-1 expression [**(B)**, red], P-selectin expression [**(C)**, red], and vWF deposition [**(D)**, red] on HMEC-1 incubated with medium alone (CTR) or with S1 (10 nM) in the presence of anti-ACE2 Ab (2 μg/ml) or Irr Ab (2 μg/ml). **(E)** Quantification and representative Western Blots of AMPK activation, evaluated as the ratio between the expression of pAMPK^Thr172^ and total AMPK in HMEC-1 exposed for 24h to medium alone (CTR) or S1 (10 nM). **(F, G)** Quantification of ICAM-1 expression **(F)** and vWF deposition **(G)** on HMEC-1 incubated with medium alone (CTR) or with S1 (10 nM) in the presence or absence of AMPK agonist AICAR (2 mM). All experiments were repeated at least 3 times. Data represent mean ± SEM and were analysed with unpaired t-test or Tukey’s multiple comparison test, as appropriate. **p-value<0.01, and ***p-value<0.001 *vs* CTR; °°°*p-value*<0.001 *vs* 10 nM S1; *^###^p-value*<0.001 *vs* 10 nM S1+Irr. All slides were counterstained with DAPI (blue). Scale bar 20 μm for **(B, C)** and 50 μm for **(D)**.

Several reports have suggested that, among the different host cell receptors, ACE2 could be the key surface protein for SARS-CoV-2 binding and entry into endothelial cells (57). Thus, we first investigated whether HMEC-1 express ACE2 and whether S1 could affect its expression. As shown in **Figure 2A**, HMEC-1 constitutively express ACE2 protein which, however, was not modulated by exposure to 10 nM S1. This finding was also confirmed in Vero cells exposed to 10 nM S1, used here as a positive control (**Supplementary Figures 2A, B** **)**.

Then, we explored whether S1 engagement with ACE2 was instrumental to the observed changes in endothelial cell phenotype. To this end, we studied whether the upregulation of adhesive and pro-thrombotic molecules induced by S1 was prevented by the inhibition of ACE2. In our setting, we observed that the upregulation of ICAM-1 induced by S1 was reduced significantly by the functional blocking antibody anti-ACE2 (ACE2) but not by an irrelevant (Irr) antibody (**Figure 2B**). Then, we studied P-selectin, an adhesive molecule that participates with ICAM-1 in endothelial cell/leukocyte interaction and in promoting pro-thrombotic processes (58, 59). We observed increased expression of P-selectin on the surface of S1-treated endothelial cells, which was inhibited significantly by ACE2 antibody (**Figure 2C**). Similarly, functional blockade of ACE2, but not an Irr Ab, significantly reduced vWF staining on the endothelial surface (**Figure 2D**). When we studied the expression of the endothelial adhesive molecule vitronectin receptor (αVβ3), we found that S1 failed to modulate its expression on endothelial cells (**Supplementary Figure 3A** **)**. Lastly, we investigated the expression of thrombomodulin, a glycoprotein that confers cytoprotective, anti-inflammatory and thromboresistant properties to endothelial cells (60, 61). We observed that S1 challenge significantly reduced thrombomodulin (CTR: 703 ± 35 *vs* 10 nM S1: 258 ± 22, pixel^2^/cell; **Supplementary Figure 3B**), which was inhibited significantly by ACE2 functional blockade (10 nM S1+ACE2: 536 ± 102 vs S1+Irr: 228 ± 47, pixel^2^/cell; representative images are shown in **Supplementary Figure 3B**).

Taken together, these data suggest that S1, by targeting ACE2, plays a critical role in inducing the activation of the microvascular endothelium, driving the shift toward a pro-inflammatory and pro-thrombotic endothelial phenotype.

### SARS-CoV-2-Derived S1 Protein Impairs AMPK Signaling

To find the molecular mechanisms behind the phenotypic changes in microvascular endothelial cells that S1 induces, we investigated whether AMPK signaling is involved (42). As shown in **Figure 2E**, we observed a significant reduction in AMPK activity in HMEC-1 upon 10 nM S1 exposure, as revealed by the decreased ratio of phospho AMPK (pAMPK) and total AMPK by Western Blot analysis. Given that AMPK plays a major role in regulating the expression of adhesive molecules (62–64), we studied the effect of the AMPK agonist AICAR on S1-treated HMEC-1. In this setting, AICAR exerted a beneficial effect on endothelial cells by reducing the expression of ICAM-1 and vWF induced by S1 (**Figures 2F, G**, and **Supplementary Figure 4A, B**), providing direct evidence that AMPK signaling plays a protective role in mediating the endothelial cell response to the S1 protein.

### SARS-CoV-2-Derived S1 Protein Induces Endothelial Dysfunction, Leading to Aberrant Complement Activation *via* ACE2

**Figure 3:**
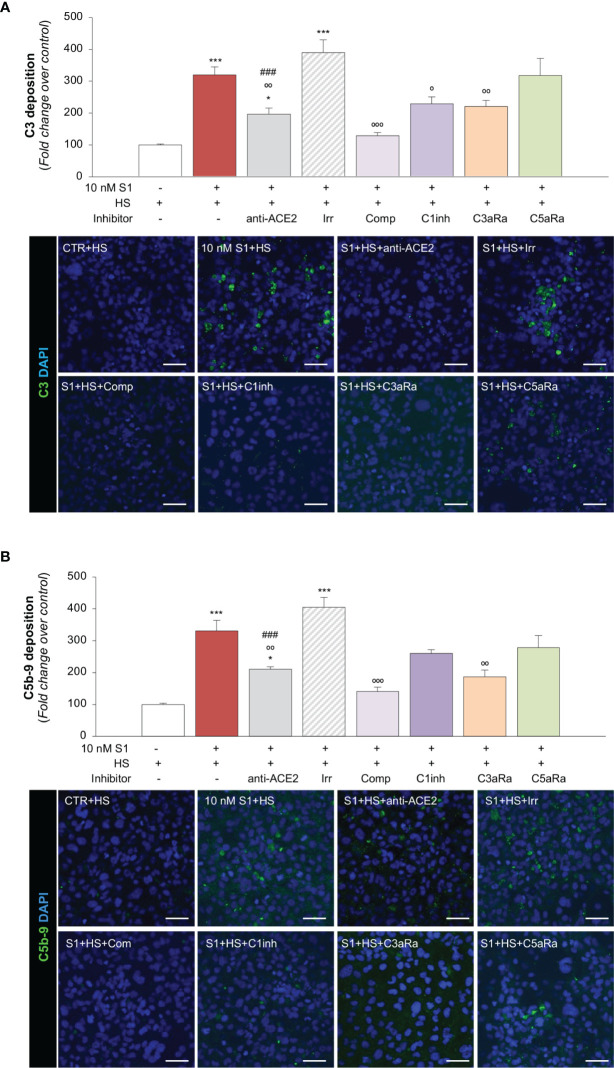
S1 induces C3 and C5b-9 deposition on HMEC-1 by interacting with ACE2. **(A)** Quantification and representative images of C3 deposition (green) on HMEC-1 pre-exposed for 24h to medium alone (CTR) or to S1 (10 nM) in the presence of anti-ACE2 Ab (2 μg/ml) or the corresponding Irr Ab (2 μg/ml) and then incubated with human serum (HS, 50%) for 2h in the presence or in the absence of complement inhibitors (Compstatin, Comp; C1 inhibitor, C1inh; C3a receptor antagonist, C3aRa; C5a receptor antagonist, C5aRa). **(B)** Quantification and representative images of C5b-9 formation (green) on HMEC-1 pre-exposed for 24h to medium alone (CTR) or S1 (10 nM) in the presence of anti-ACE2 Ab (2 μg/ml) or Irr Ab (2 μg/ml) and then incubated with HS 50% in the presence or in the absence of complement inhibitors (Comp, C1inh, C3aRa, and C5aRa). All experiments were repeated at least 3 times. Data represent mean ± SEM and were analysed with Tukey’s multiple comparison test. *p-value<0.05, and ***p-value<0.001*vs* CTR; °*p-value*<0.05, °°*p-value*<0.01, and °°°*p-value*<0.001 *vs* 10 nM S1; *^###^p-value*<0.001 *vs* 10 nM S1+Irr. All slides were counterstained with DAPI (blue). Scale bars 50 μm.

Recent evidence suggests that the complement system may play a substantial role in promoting microvascular inflammation, which appears to contribute to the severity of COVID-19 (65–67). The finding that the upregulation of adhesive molecules, such as P-Selectin (58, 59), or the loss of thrombomodulin (59, 68), may drive the complement attack on the endothelium, prompted us to investigate whether S1 protein-induced endothelial dysfunction could modulate complement activation and deposition on the microvascular endothelium. To this end, we exposed HMEC-1 to S1 and then to human serum (HS) from a pool of healthy volunteers as a source of complement. As shown in **Figure 3A**, we observed massive C3 deposition on HMEC-1 exposed to 10 nM S1, compared to unstimulated cells. When endothelial cells were exposed to S1 in the presence of anti-ACE2 Ab, there was a significant decrease in C3 staining, compared to the deposits observed on cells treated with an Irr Ab (**Figure 3A**), demonstrating that the S1/ACE2 axis plays a key role in mediating complement activation at the endothelial level. Pre-treating human serum with complement C3 inhibitor compstatin led to a significant decrease in C3 staining that was comparable to that of unstimulated HMEC-1 (**Figure 3A**). A significant inhibitory effect was found in the presence of complement 1 inhibitor, which blocks the classical pathway (**Figure 3A**). Notably, blocking the endothelial C3a receptor with a specific antagonist led to a significant decrease in C3 deposits on endothelial cells, whereas C5a receptor blockade had no effect (**Figure 3A**).

In our assay, we also demonstrated that complement activation on HMEC-1 exposed to 10 nM S1 protein proceeded to the formation of the terminal membrane attack C5b-9 complex, as shown in **Figure 3B**. The massive C5b-9 staining that had been observed on endothelial cells exposed to S1 protein was largely prevented by anti-ACE2 Ab but not by Irr Ab (**Figure 3B**). Blocking C3 activation with compstatin led to a profound inhibition of C5b-9 formation on the surface of HMEC-1 (**Figure 3B**). The partial effect of C1 inhibitor on C5b-9 staining was like that obtained on C3 deposits (**Figure 3B**). Blockade of the C3a and C5a receptors led to an inhibition of C5b-9 formation on HMEC-1 (**Figure 3B**).

Overall, these data indicate that endothelial dysfunction induced by SARS-CoV-2-derived S1 protein triggers exuberant complement deposition on activated microvascular endothelial cells and that the anaphylatoxins C3a and, to a lesser extent, C5a, further amplify the complex process of complement activation that fuels inflammation in response to S1.

### SARS-CoV-2-Derived S1 Protein Induces Endothelial Dysfunction, Promoting Leukocyte Adhesion *via* ACE2

**Figure 4:**
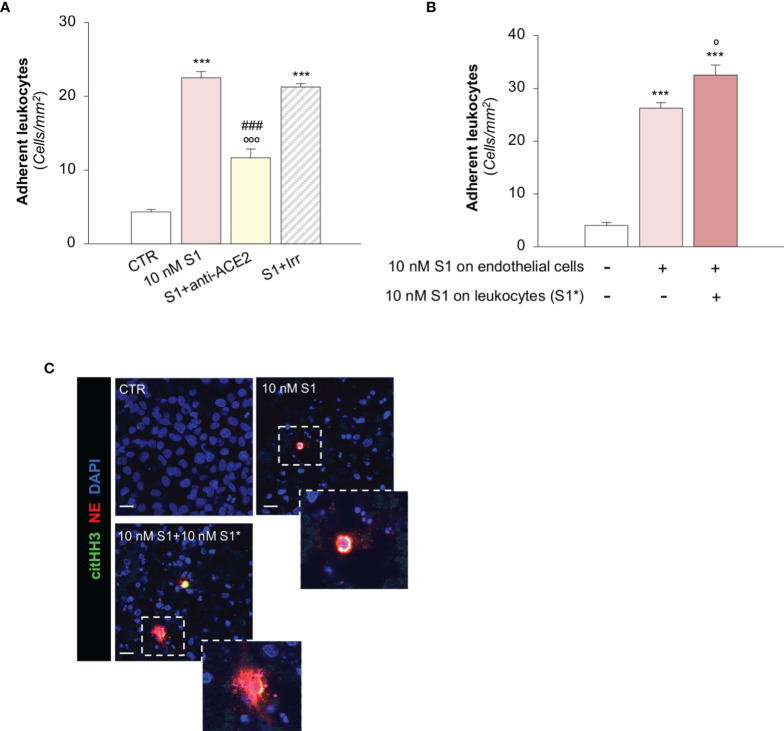
S1 promotes leukocyte adhesion and NET formation on HMEC-1 under flow. **(A)** Quantification of leukocyte adhesion under flow conditions on HMEC-1 exposed for 24h to medium alone (CTR) or to subtoxic concentration of S1 (10 nM) in the presence of anti-ACE2 functional blocking Ab (ACE2, 2 μg/ml) or the corresponding Irr Ab (2 μg/ml). **(B)** Adhesion of leukocytes, incubated for one hour with control medium or with S1 (S1*, 10 nM) and perfused under flow conditions (1.5 dynes/cm^2^) on HMEC-1 exposed to medium alone (CTR) or with S1 (10 nM). **(C)** Representative images of leukocytes treated with medium alone (CTR) or S1 (S1*, 10 nM), which adhered to HMEC exposed for 24h to medium alone or to S1. In this setting, neutrophils were co-stained with histone H3 citrullinated (citHH3, green) and neutrophil elastase (NE, red). The release of neutrophil extracellular traps (NETs) was observed only when leukocytes were activated with S1 (10 nM S1+ 10 nM S1*, inset). All experiments were repeated at least 3 times. Data represent mean ± SEM and were analysed with Tukey’s multiple comparison test. ***p-value<0.001 *vs* CTR; *°p-value*<0.05, and °°°*p-value*<0.001 *vs* 10 nM S1; *^###^p-value*<0.001 *vs* 10 nM S1+Irr. Slides were counterstained with DAPI (blue). Scale bar 20 μm.

To investigate the functional impact of the observed endothelial phenotypic changes, we first determined whether the activation of HMEC-1 promoted by S1 was instrumental to the recruitment of leukocytes under flow conditions. Exposure of HMEC-1 to 10 nM S1 markedly induced leukocyte adhesion on HMEC-1 in a parallel plate flow chamber (**Figure 4A**). This effect was like that obtained by a positive control such as Shiga Toxin 2 (Stx2, **Supplementary Figure 5A**). Data that the functional blocking antibody against ICAM-1 robustly reduced the number of adherent leukocytes (**Supplementary Figure 5B**) confirmed that the upregulation of ICAM-1 on S1-activated HMEC-1 was accountable for leukocyte stable adhesion. We also found that ACE2 affected the adhesive properties of S1-activated endothelial cells, as demonstrated by the significant decrease in leukocyte adhesion by ACE2 functional blocking antibody, unlike with the Irr antibody (**Figure 4A**). Pre-exposure of leukocytes to 10 nM S1 resulted in more adherent leukocytes on S1-treated HMEC-1 (**Figure 4B**). Co-staining of histone H3 citrullinated (citHH3, green) and neutrophil elastase (NE, red) revealed that neutrophils adhered to S1-treated HMEC-1 and were activated to release NE in extracellular traps (NETs, inset) when they were challenged with 10 nM S1 (**Figure 4C**, insets). No detectable NETs were found when unstimulated leukocytes were perfused on S1-activated HMEC-1 (**Figure 4C**, insets).

A recent study has suggested that aberrant complement activation during COVID-19 plays an important role in the activation of circulating neutrophils (65). Therefore, we proceeded to evaluate whether C3a generated during the S1-induced C3 activation amplifies the process of leukocyte-endothelial interaction induced by S1. Notably, we found that the number of adherent S1-treated leukocytes on S1-activated HMEC-1 rose significantly after endothelial exposure to C3a, compared to endothelial cells exposed to S1 alone (adhesion of S1-treated leukocytes on: 10 nM S1-treated HMEC-1, 35 ± 2 *vs* 10 nM S1- and C3a-treated HMEC-1, 46 ± 2, adherent leukocytes/mm^2^).

### SARS-CoV-2-Derived S1 Protein Induces Platelet Aggregates, Exacerbated by Complement Activation, on Microvascular Endothelial Cells

**Figure 5:**
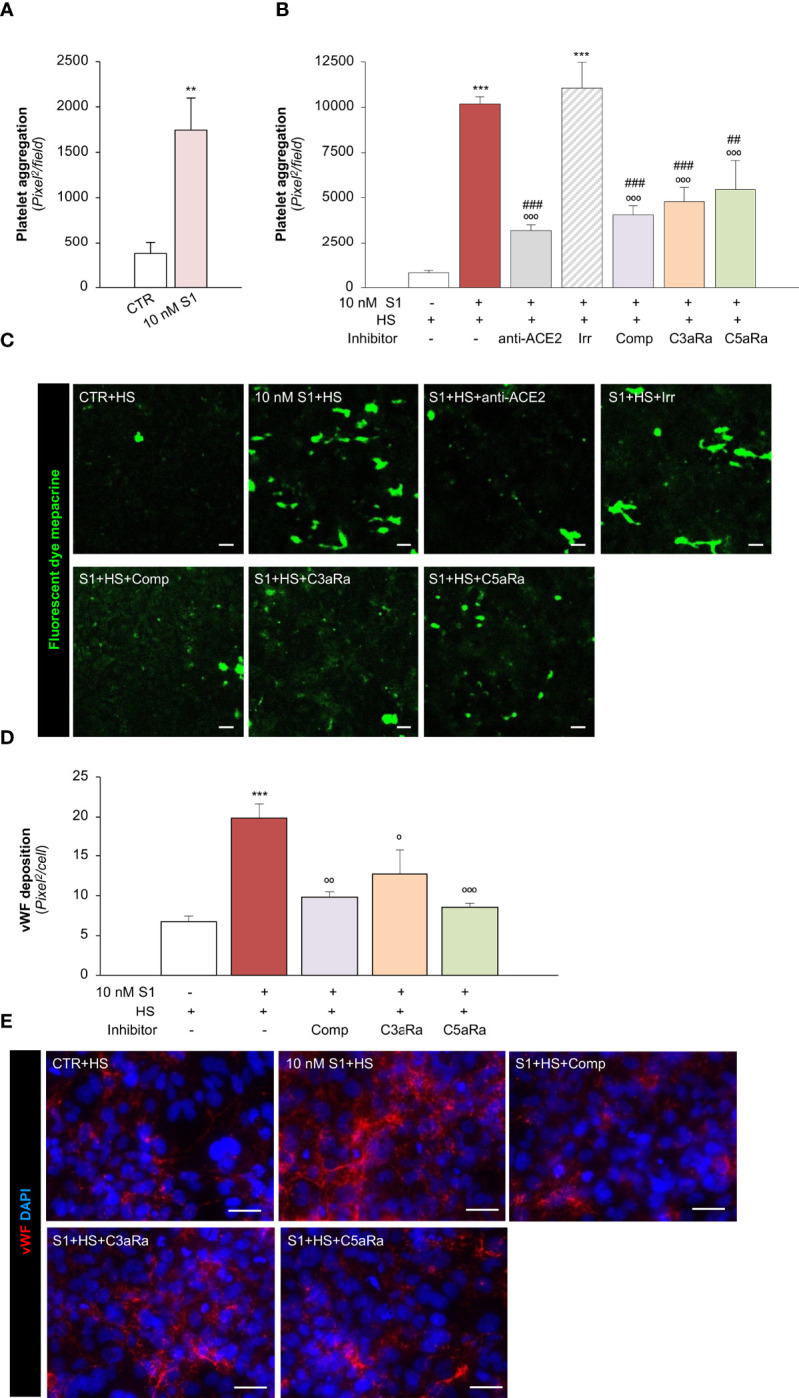
S1 activates the complement system amplifying platelet aggregate formation on HMEC-1 through ACE2. **(A)** Quantification of platelet aggregate formation on HMEC-1 pre-exposed for 24h to medium alone (CTR) or S1 (10 nM). Platelets aggregates on HMEC-1 perfused with heparinised blood under flow conditions (60 dynes/cm^2^) were evaluated and expressed as pixels^2^/field analysed. **(B, C)** Quantification **(B)** and representative images **(C)** of platelet aggregate formation on HMEC-1 pre-exposed for 24h to medium alone (CTR), S1 (10 nM), or 10 nM S1 in the presence of anti-ACE2 Ab (2 μg/ml) or the corresponding Irr Ab (2 μg/ml) and then incubated with 50% human serum (HS) for 2h. In selected samples, S1-treated HMEC-1 were incubated with 50% HS in the presence of complement inhibitors (Compstatin, Comp; C3a receptor antagonist, C3aRa; C5a receptor antagonist, C5aRa). Platelet aggregate formation on HMEC-1 under flow conditions (60 dynes/cm^2^) was quantified and expressed as pixel^2^/field analysed. **(D, E)** Quantification **(D)** and representative images **(E)** of vWF deposition on HMEC-1 pre-exposed for 24h to medium alone (CTR) or S1 (10 nM) and then incubated with 50% HS in the presence or absence of complement inhibitors (Comp, C3aRa, and C5aRa). All experiments were repeated at least 3 times. Data represent mean ± SEM and were analysed with unpaired t-test or Tukey’s multiple comparison test, as appropriate. **p-value<0.01, and ***p-value<0.001 *vs* CTR; °*p-value*<0.05, °°*p-value*<0.01, and °°°*p-value*<0.001 *vs* 10 nM S1; *^##^p-value*<0.01 and *^###^p-value*<0.001 *vs* 10 nM S1+Irr. Scale bars 50 μm.

We then moved on to evaluating whether the endothelial dysfunction induced by the S1 protein also has functional relevance in disrupting the thromboresistant phenotype of the microvascular endothelium. To this end, we perfused heparinized whole blood on S1-activated HMEC-1 in a parallel plate flow chamber. Our data showed that 10 nM S1 exposure on HMEC-1 promoted significant platelet deposition and aggregation on the cell surface compared to unstimulated cells under flow (**Figure 5A**). The S1-induced platelet aggregate formation was comparable to that observed following Stx2 exposure (Stx2: 2277 ± 129 pixel^2^/field), used as a positive control (69).

To demonstrate how complement activation contributes to amplifying S1-dependent platelet aggregation, S1-treated HMEC were exposed to HS as a source of complement. Here, we found that when perfused with whole blood under flow conditions (**Figures 5B, C** **)**, HMEC-1 exposed to 10 nM S1 protein exhibited a more than 10-fold increase in the cell area covered by platelet aggregates compared to unstimulated cells. The addition of anti-ACE2 Ab during incubation with 10 nM S1 protein almost normalized platelet aggregate formation on the endothelial cell surface, which remained unaffected after the addition of Irr Ab (**Figures 5B, C** **)**. The pivotal role of the complement system was demonstrated by the remarkable inhibition of platelet aggregation detected on 10 nM S1-treated HMEC-1 in the presence of HS incubated with C3 inhibitor compstatin, C3aR or C5aR antagonists, respectively (**Figures 5B, C** **)**.

We then evaluated whether complement activation affects vWF deposition on HMEC-1. In this experimental setting, exposure of endothelial cells to 10 nM S1 and HS also induced a significant increase in vWF on the endothelial cell surface (**Figures 5D, E** **)**. Conversely, the addition to HS of the C3 inhibitor compstatin, C3aR, or C5aR antagonists, significantly decreased vWF staining on HMEC-1 exposed to 10 nM S1 (**Figures 5D, E** **)**.

Overall, these data indicate that S1 promotes a robust microvascular endothelial cell response, which is a determinant in the propagation of pro-inflammatory and thrombogenic processes both amplified by the activation of the complement system.

### SARS-CoV-2-Derived S1 Activates Pulmonary Endothelial Cells to Express Adhesive and Pro-Thrombotic Molecules by Binding to ACE2

**Figure 6:**
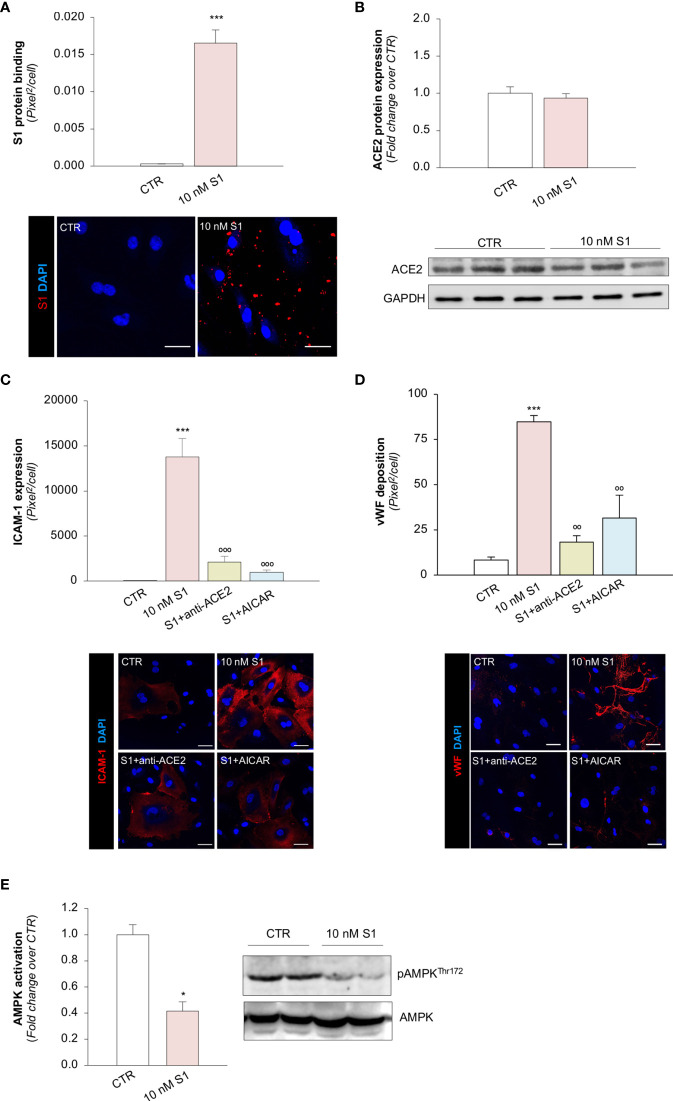
S1, through ACE2, upregulates adhesive molecules on HPMEC by impairing AMPK signalling. **(A)** Quantification and representative images of the binding of the S1 protein (red) to HPMEC treated with medium alone (CTR) or S1 (10 nM) for 24 h. **(B)** Quantification and representative Western Blots of ACE2 protein expression in HPMEC exposed for 24h to medium alone (CTR) or S1 (10 nM). GAPDH was used as a sample loading control. **(C, D)** Quantification and representative images of ICAM-1 expression [**(C)**, red], and vWF deposition [**(D)**, red] on HPMEC incubated with medium alone (CTR) or with S1 (10 nM) in the presence or absence of anti-ACE2 Ab (2 μg/ml) or AICAR (2 mM). **(E)** Quantification and representative Western Blots of AMPK activation, evaluated as the ratio between the expression of pAMPK^Thr172^ and total AMPK in HPMEC exposed for 24h to medium alone (CTR) or 10 nM S1. All experiments were repeated at least 3 times. Data represent mean ± SEM and were analysed with unpaired t-test or Tukey’s multiple comparison test, as appropriate. *p-value<0.05, and ***p-value<0.001 *vs* CTR; °°*p-value*<0.01, and °°°*p-value*<0.001 *vs* 10 nM S1. All slides were counterstained with DAPI (blue). Scale bar 50 μm.

To provide proof-of-concept that the above mechanisms are also shared by microvascular endothelial cells in the lungs, we further studied the effect of S1 on the expression of ACE2, ICAM-1, vWF and AMPK signalling on primary human pulmonary microvascular endothelial cells (HPMECs). First, we found that S1 bound to the surface of HPMEC (**Figure 6A**). We confirmed that HPMEC constitutively express ACE2, whose expression was not affected by S1 challenge **(** **Figure 6B**). Notably, we found that HPMEC expressed ACE2 to a similar extent as Vero cells, used as a positive control, while they expressed higher ACE2 levels than HMEC-1 (**Supplementary Figure 6**). Exposure of HPMEC to S1 elicited a marked increase in ICAM-1 and vWF stainings, which were limited by the addition of ACE2 blocking antibody (**Figures 6C, D** **)**. Lastly, we found that S1 impaired AMPK signalling (**Figure 6E**). The finding that AICAR prevented ICAM-1 and that vWF increased expression induced by S1 (**Figures 6C, D** **)** demonstrated that AMPK signalling plays a critical, protective role in HPMEC activation.

All these data suggest that S1 induced a robust response in HPMEC, even more than HMEC-1, and caused a loss in the thromboresistant phenotype *via* ACE2.

## Discussion

In this study, we describe a mechanism that has never been reported through which SARS-CoV-2-derived S1 protein alone induces a pro-inflammatory and pro-thrombotic phenotype of human microvascular endothelial cells of dermal and pulmonary origin, possibly recapitulating the systemic microvascular complications observed in severe COVID-19 cases.

The first finding of this study is that the spike protein of SARS-CoV-2 can be found in the sera of patients with COVID-19. Indeed, using ELISA we found that spike protein is detected in at least 30% of hospitalized patients with severe disease. Although this assay cannot discriminate between intact SARS-CoV-2 virus and free spike protein, a recent study suggests that SARS-CoV-2 can be found in the circulation in a similar proportion (30-40%) of severe COVID-19 patients and that plasmatic viremia correlates with disease severity and mortality (34). Our finding that patients positive for plasmatic spike protein had a borderline significant increase in the rate of SARS-CoV-2 RNA positivity suggests that spike protein-positive patients may have had a higher viral load at the time of hospitalization than spike protein-negative subjects. However, we cannot exclude the possibility that the presence of spike protein could be the result of systemic viral particle release during COVID-19. At this stage of the disease, elevated viremia could contribute to the diffusion of viral particles that may damage microvascular endothelial cells in the lungs and in more distal organs, leading to systemic thrombotic complications. In line with this possibility, a study in mice showed that circulating S1 could localise in the microvascular endothelium (54). Furthermore, a recent report documented that circulating nucleocapsid protein can be detected in the sera of COVID-19 positive subjects, particularly in those who remain negative for anti-SARS-CoV-2 antibodies, suggesting that systemic shedding of viral components likely occurs soon after infection (70). However, our serological analysis was performed on the sera of hospitalized patients, who are generally admitted to the hospital 10 days after symptom onset (32, 49–51). All available data regarding the kinetics in SARS-CoV-2 viral load reveal that the viraemic peak occurs early, 2-4 days after infection, and viral shedding is almost absent 10 days after infection (32, 49–51). In line with this finding, we found that patients positive for plasmatic spike protein tended to be admitted earlier after symptom onset. The presence of spike protein in the sera of hospitalized patients suggests that the total burden of circulating S1 on endothelial cells could be significantly higher early during COVID-19.

Based on our data and the available published studies, we can infer that, during the early phase of the infection, the lung microvasculature is the first target of SARS-CoV-2. Indeed, the high replication rate of the virus in lung epithelial cells could particularly affect the local microvascular endothelial cells lining the capillaries in the alveoli. In this phase, pulmonary embolism has been identified as the hallmark feature of microvascular thrombosis in severe COVID-19 cases (71, 72). As the disease progresses, sustained shedding of viral protein may target microvascular endothelial cells in organs distal to the lungs, possibly leading to systemic thrombotic complications (73).

Following these observations, here we investigated whether S1 protein could induce endothelial cell activation and dysfunction to understand the underlying mechanisms that may recapitulate the microvascular thrombotic complications observed in COVID-19. We provide data that S1 alone was able to activate human microvascular endothelial cells, thus promoting leukocyte adhesion. The ability of S1 to induce inflammatory cell recruitment on endothelial cells was the result of the upregulation of endothelial P-selectin and ICAM-1, which are known to be key players in the paradigm of rolling and stable adhesion of inflammatory cells on the endothelium (55).

To demonstrate that the S1 directly elicits cell signalling *via* interaction with its cognate receptor, we showed that the functional blockade of ACE2 was sufficient to inhibit S1-dependent increased endothelial adhesiveness to leukocytes. The hypothesis that ACE2 plays a fundamental role in S1-endothelial cell interaction is supported by the available data, which confirms that soluble human recombinant ACE2 halted SARS-CoV-2 infection in engineered human blood vessel organoids (74). However, we cannot rule out the possibility that other endothelial receptors, such as neuropilin-1 (75), dipeptidyl peptidase 4 (76), and CD147 (77) contribute to S1-induced endothelial cell injury.

Although it is important to consider that S1 can induce exocytosis from Weibel-Palade bodies, contributing to increased expression of P-selectin on endothelial cells, it is possible that the engagement of S1 with ACE2 activates endothelial gene transcription of P-selectin and ICAM-1. Our *in vitro* studies support this hypothesis because they clearly demonstrated that S1-ACE2 interaction impaired AMPK signalling, inducing an increase in the adhesive properties of endothelial cells. Indeed, AMPK inhibits NF-κB (78), the main transcription factors involved in the expression of proinflammatory and adhesive proteins (79). Notably, the importance of AMPK signalling is a common feature of the microvascular endothelial cells of different origin.

Our data also revealed an additional mechanism through which S1 potentiates microvascular endothelial injury *via* the direct activation of neutrophils. Indeed, leukocytes challenged with S1 further increased their recruitment on S1-activated endothelial cells and promoted neutrophil NET release. The clinical relevance of our data rests on the findings that both the accumulation of neutrophils activated by SARS-CoV-2 (80), and the formation of NETs in damaged tissue are associated with a poor COVID-19 prognosis (81, 82). Collectively, our findings highlight that S1 plays a direct role in fuelling the process of inflammation in microvascular endothelial cells.

Exposing HMEC-1 to S1 significantly promoted the deposition of platelet aggregates under flow at high shear stress, which demonstrates that S1 directly affects the endothelial thromboresistant phenotype. This phenomenon was likely the result of the S1-induced alterations in the complex interplay between the surface expression of P-selectin and vWF, due to increased exocytosis. However, the contribution of blood-derived vWF should also be considered, as revealed by the lower level of vWF expression in the serum-free experimental setting. It is well known that endothelial P-selectin participates in the process of thrombosis by binding directly to platelets or by interacting with vWF, which further supports our data. At high shear stress, this latter fundamental adhesive substrate enables the deposition of platelets, tethering their GPIb and then αIIβ3 receptors (56, 69).

Furthermore, the ability of S1 to induce the loss of thrombomodulin – a cofactor that prevents local fibrin formation and that is also an inhibitor of complement activation (59) – may amplify the thrombotic effects triggered by the viral protein. Considering that S1 was able to promote exocytosis in endothelial cells, the loss of thrombomodulin could be the result of its shedding by newly exposed proteases on the cell surface.

Our *in vitro* study highlights the direct role that S1-ACE2 interaction plays in engaging intracellular signalling, contributing to the impairment of vascular integrity and development of a pro-thrombotic state that may have pathophysiological implications in COVID-19.

Earlier studies have shown that alterations in the endothelial thromboresistant phenotype, including the overexpression of endothelial P-Selectin (58, 69) and the loss of thrombomodulin (59, 68), contribute to complement activation, which increases the risk of thrombosis. In line with these reports, we have provided evidence that S1 interaction with ACE2 led to marked C3 and C5b-9 deposition on endothelial cells, which was associated with increased formation of platelet aggregates. The complement system plays a major role in exacerbating this phenomenon, as confirmed by data that showed that the specific C3 inhibitor compstatin, as well as the inhibitors of C3a and C5a receptors, robustly inhibited platelet deposition on S1-activated endothelial cells. Furthermore, several pieces of evidence support the hypothesis that the terminal complement pathway plays a role in exacerbating the inflammatory reaction on the endothelium by promoting neutrophil and macrophage recruitment and their activation to generate an oxidative burst (83). Additionally, C3a and, to a lesser extent, C5a – generated following S1 exposure – were the key mediators in the amplification of the complement cascade and, together with C3, contributed to S1-dependent microvascular thrombosis. There is evidence that C3a and C5a, generated following complement activation, are driving factors in altering endothelial thromboresistance (59, 84, 85). Further proof-of-concept that C5a has thrombogenic effects on endothelial cells comes from data showing that C5a inhibition halts the platelet aggregation induced by sera from severe COVID-19 patients, possibly through the exocytosis of vWF and P-selectin (48). Finally, data from the UK show that genetic predisposition to complement dysregulation is a risk factors for morbidity and death from SARS-CoV-2 infection, which indicates that hyperactivation of complement is a hallmark feature of the pathophysiology of severe COVID-19 (86, 87).

Based on the above, inhibiting the complement system could be a potential treatment for COVID-19 patients. A recent case report on a patient with ARDS due to COVID-19 pneumonia showed that treatment with a C3 inhibitor was safe and associated with a favourable outcome (88). Much larger case series have shown that the C5 inhibitor, eculizumab, and a MASP-2 inhibitor, narsoplimab, may have also some therapeutic efficacy (89–91).

Limitation of the study: one of the major limitations of our study is the small sample size of patients, which may affect the analysis outcomes. Additionally, the analysis of circulating spike protein was performed exclusively in serum from COVID-19 patients obtained at the time of hospital admission, making it impossible to study the early phase kinetics of circulating spike proteins, as well as the temporality of the thrombotic phenomena in COVID-19. Furthermore, these patients were in an advanced phase of the disease and thrombosis was mainly diagnosed based on the analysis of medical records. Additionally, thrombosis in COVID-19 is often difficult to detect, particularly in mechanically ventilated patients with severe pneumonia. Finally, the ELISA assay used to detect the spike protein in human sera cannot differentiate between intact SARS-CoV-2 virus and free spike protein.

In summary, our study documented that: 1) circulating spike protein can be found in severe COVID-19 patients; 2) S1 directly induced the activation of multifaceted deleterious processes that lead to endothelial cell dysfunction; 3) engagement of ACE2 by S1 is sufficient to alter the adhesive properties of microvascular endothelial cells by altering AMPK signalling, resulting in the recruitment of inflammatory cells; 4) the S1-induced inflammatory phenotype favours exuberant C3 and C5b-9 deposits on microvascular endothelial cells, and the generation of C3a and C5a further amplified the complement activation induced by S1; 5) all these events promote a loop of reciprocal activation, ultimately leading to increased platelet aggregates on microvascular endothelial cells.

Overall, these data provide novel insights that can help to identify more effective therapies to inhibit the complement system in patients with severe COVID-19.

## Materials And Methods

### Ethics Statement

Sera from convalescent COVID-19 patients with mild disease or negative subjects were selected from a previous study by our group in the same geographical area and during the same period of time (92). These subjects were selected in order to obtain age- and sex-matched controls for the severe COVID-19 cases. Patients with severe COVID-19 who were admitted to the COVID Unit of the Azienda Socio Sanitaria Territoriale (ASST) Papa Giovanni XXIII hospital in Bergamo (Italy) between March and June 2020 because of severe respiratory distress due to COVID-19 diagnosed on the basis of the 19 March 2020 WHO Interim guidance criteria (93). Sera from severely ill COVID-19 patients were collected at hospital admission. The study was approved by the Ethical Committee of the Azienda Sanitaria Locale Bergamo, Italy. Written informed consent was obtained from all enrolled patients. All patients’ characteristics are summarized in **Table 1**.

### Detection of Spike Protein and Complement Components in Human Samples

To detect SARS-CoV-2 spike protein in human sera, the specific COVID-19 Spike Protein ELISA Kit (Abcam, ab274342) was used, following the manufacturer’s instructions. Briefly, microwell plates were coated with SARS-CoV-2-derived spike antibody and incubated with human sera. Antigen detection was performed by incubation with an anti-SARS-CoV-2 spike antibody, followed by streptavidin-HRP conjugate. Measurement of OD at 450 nm was performed on the multimode microplate reader TECAN Infinite M200^®^ PRO.

Plasmatic levels of sC5b-9 and C5a were evaluated using MicroVue sC5b-9 Plus EIA (Quidel) and MicroVue C5a EIA (Quidel). Blood was collected in ice-cold EDTA tubes and immediately centrifuged at 4°C to avoid *ex vivo* complement activation. Plasma was quickly separated and frozen at -80°C until assay.

### Endothelial Cell Cultures and Experimental Design

A large body of the literature reported the use of different types of immortalized endothelial cell lines to study SARS-CoV-2 infection (47, 94–96). We chose to study the human microvascular endothelial cell line of dermal origin (HMEC-1; RRID: CVCL_YJ39), obtained from Dr Edwin Ades and Francisco J. Candal (Centers for Disease Control and Prevention) and Dr Thomas Lawley from Emory University (97). Cells were cultured and validated as described previously (69). In our setting, HMEC-1, obtained from different batches, were used at low passages (max 20^th^) in order to retain all the endothelial functions during culture (69).

For cell viability studies, HMEC-1 were exposed to MCDB 131 medium (Invitrogen) supplemented with 2% Fetal Calf Serum (FCS, Invitrogen) in the presence or absence of SARS-CoV-2-derived spike protein 1 (S1) at a concentration of 0.5 nM (37.5 ng/ml), 10 nM (750 ng/ml), and 50 nM (3750 ng/ml) for 24 h and then a cell count was performed. The S1 used for all the experiments is a commercially available recombinant SARS-CoV-2 S1 purified by metal ion affinity chromatography (230-01101, RayBiotech). The above range of concentrations was chosen on the basis of previous studies (47, 53, 54).

All the experimental designs are summarized in **Supplementary Figures 7A, B**. For the leukocyte adhesion assay, leukocyte suspensions were incubated for 1h with control medium or S1 (10 nM) before perfusion on unstimulated or S1-treated HMEC-1 (24 h, 10 nM). In selected experiments, HMEC-1 were incubated with S1 for 24 h in the presence of anti-ACE2 functional blocking antibody (Ab, 2 μg/ml, Adipogen, AG-20A-0037PF), anti-ICAM-1 functional blocking Ab (10 μg/ml, Merck, MAB2146), or the corresponding irrelevant (Irr) Ab (IgG mouse, Santa Cruz, sc2025) at the proper concentration (2 and 10 μg/ml) 1 h before S1 incubation (**Supplementary Figure 7A**). In additional experiments, HMEC-1 were incubated with 1 μM C3a (A118, Complement Technology; for 4 h) or 50 pM Shiga Toxin 2 (Stx2, for 24 h) – used here as a positive control (98) – before leukocyte perfusion.

For immunofluorescence studies, HMEC-1 were incubated for 24h with control medium or S1 (10 nM) in the presence or absence of anti-ACE2 Ab (2 μg/ml, Adipogen) or the corresponding Irr Ab (IgG mouse, Santa Cruz). In additional experiments, after 24 h incubation with control medium or S1, cells were exposed for 2 hours to a pool of human serum from healthy donors (HS) diluted 1:2 with test medium (HBSS with 0.5% BSA) in the presence or in the absence of compstatin (100 µM, Tocris bioscience, 2585/1) to block the complement component C3, or complement inhibitor 1 (36 µg/ml, Merck, SRP3318) to block the classical complement pathway. In this setting, the addition of a C3a receptor antagonist (1 µM, Merck, 559410) or a C5a receptor antagonist (10 µM, Merck, 234415) were tested (**Supplementary Figure 7B**). In additional samples, AMPK activator, 5-aminoimidazole-4-carboxamide-1-β-D-ribofuranoside (AICAR, Toronto Research Chemicals Inc, A611700) was used at a concentration of 2 mM.

For the analysis of vWF localization, HMEC-1 were stained with 1 μM cell tracker green CMFDA (Life Technology, C7025) for 30 minutes at 37°C at the end of incubation with control medium or 10 nM S1.

For the platelet adhesion assay under flow conditions, HMEC-1 were incubated for 24h with control medium or S1 (10 nM) before blood perfusion. In additional experiments, after 24h of incubation with medium or S1, HMEC-1 were exposed for 2 hours to HS diluted 1:2 in the presence or in the absence of anti-ACE2 Ab (2 μg/ml, Adipogen) or the corresponding Irr Ab (IgG mouse, Santa Cruz), or C3 inhibitor compstatin (100 µM, Tocris bioscience). In this setting, a C3a receptor antagonist (1 µM, Merck) or a C5a receptor antagonist (10 µM, Merck) were also added in HS diluted 1:2.

Human primary pulmonary microvascular endothelial cells (HPMEC; Lonza, CC-2527) were grown in EGM™-2 MV Microvascular Endothelial Cell Growth Medium-2 BulletKit™ (Lonza, CC-3202) following the manufacturers’ instructions. According to the manufacturer’s validation, HPMEC express CD31/105, von Willebrand Factor VIII, are positive for acetylated low density lipoprotein uptake, and are PECAM positive. HPMEC were incubated with S1 at a concentration of 10 nM for 24h in the presence or absence of 2 μg/ml anti-ACE2 Ab or 2 mM AICAR.

As a positive control for the determination of S1 binding and ACE2 expression, Vero CCL-81 cells (ATCC, CCL-81; RRID: CVCL_0059) were cultured in Eagle’s minimal essential medium (EMEM, Sigma-Aldrich) supplemented with 10% heat-inactivated fetal bovine serum (FBS) and 1% penicillin/streptomycin (P/S, Invitrogen).

### Crystal Violet Viability Assay

HMEC-1 were seeded 10000 cells/well in 96-well plates in MCDB 131 in the presence of 2% FCS and, when confluent, were exposed for 24 h to different concentrations of S1 (0.5 nM, 10 nM and 50 nM). At the end of incubation, cells were fixed and stained with 0.5% crystal violet in 20% methanol. The stain was eluted with a 1:1 solution of ethanol and 0.1M sodium citrate, the absorbance was measured at 595 nm on the multimode microplate reader (Victor3, 1420 Multilabel counter, PerkinElmer). Cell viability was evaluated as live cells stained with crystal violet after subtraction of baseline absorbance. Data are expressed as percentage of viable cells.

### Leukocyte Adhesion Assay Under Physiologic Flow Conditions

Leukocytes were isolated from blood collected on EDTA (final concentration 5 mmol/L) as we previously described (98). For adhesion experiments, we used a parallel-plate flow chamber connected to a perfusion system (98). HMEC-1 slides were flowed with leukocyte suspension (10^6^ cells/ml) with a shear stress of 1.5 dynes/cm^2^ to reproduce the circulation of post-capillary venules for 10 minutes (98). Images of adhering leukocytes on the HMEC-1 surface were acquired during the perfusion experiments, digitized, and processed using Image J software. The number of adherent leukocytes was determined on a series of 16 consecutive images. Adherent leukocytes were identified and counted at the end of the 10 min perfusion (98).

### Platelet Adhesion Assay Under Flow Conditions

Perfusion of heparinized whole blood (10 UI/ml) obtained from healthy subjects (prelabelled with the fluorescent dye mepacrine, 10 μM) was performed in a flow chamber at 60 dynes/cm^2^, as encountered in the microvasculature (59, 69). After 3 min of perfusion, the endothelial cell monolayer was fixed in acetone. Fifteen fields – systematically digitalized *per* sample of platelets, deposited along the endothelial surface – were acquired using an inverted confocal laser microscope (Leica TCS SP8, Leica Microsystems), and areas occupied by platelet aggregates were evaluated using Image J software and expressed as pixel^2^ *per* field analysed. For each sample, after excluding the lowest and the highest value, the mean was calculated based on the remaining 13 fields.

### Immunofluorescence Analysis

The slides were fixed with 3% paraformaldehyde (Società Italiana Chimici). After blocking, cells were incubated with the specific antibodies: mouse anti-SARS-CoV-2 RBD (1:1000, Abcam, ab277624), mouse anti-P-selectin (1:10, R&D Systems, BBA30), mouse-anti ICAM-1 (1:100, Merck, MAB2146), FITC-conjugated rabbit anti-C3c-complement (1:300, Dako, F0201), rabbit anti-complement C5b9 complex (1:200, Calbiochem, 204903), rabbit anti-vWF (10 μg/ml, Dako, A0082), mouse anti-thrombomodulin (1:50, R&D Systems, MAB3947) followed by the corresponding secondary antibodies (Jackson ImmunoResearch Laboratories). Nuclei were counterstained with 4’,6-diamidino-2-phenylindole (DAPI, Sigma-Aldrich). Digital images were acquired using an inverted confocal laser microscope (Leica TCS SP8) or ApoTome Axio Imager Z2 (Zeiss).

The quantification of S1 deposition on endothelial cells was performed by analysing 10 fields/sample using Image J software and expressed as the area covered by the fluorescence *per* cell number (pixel2/cell).

The quantification of endothelial P-selectin and ICAM-1 stainings was performed by analysing 10 fields/sample using Image J software and expressed as the area covered by the fluorescence *per* cell number (pixel^2^/cell).

For C3, and C5b-9, fifteen fields, systematically digitized along the surface, were acquired using a computer-based image analysis system. The area occupied by the fluorescent staining was evaluated by automatic edge detection, using built-in specific functions of Image J software and expressed as pixel^2^ *per* field analysed. vWF was quantified as above and expressed as pixel^2^ *per* cell. For each sample, after excluding the lowest and the highest values, the mean was calculated on the remaining 13 fields.

NET formation, at the end of the leukocyte adhesion assay, was studied on HMEC-1 fixed with 3% paraformaldehyde, and permeabilised with 0.1% Triton X-100 (Sigma-Aldrich, T8787). After blocking, cells were incubated with rabbit anti-Histone H3 (citrulline R2+R8 R17, 1:100, Abcam, ab5103) and mouse anti- Neutrophil Elastase (1:100, Abcam, ab254178), followed by the corresponding secondary antibodies. Nuclei were counterstained with DAPI.

### Protein Extraction and Western Blot Analysis

HMEC-1, Vero CCL-81, and HPMEC were sonicated in CelLytic M (Sigma-Aldrich, C2978) supplemented with protease inhibitor cocktail (Sigma-Aldrich, P8340). Following centrifugation at 16000xg for 10 minutes at 4°C, lysates were collected and total protein concentration was determined using DC™ assay (Bio-Rad Laboratories, 5000112).

Equal amounts of proteins (30 μg) were separated on 12% SDS-PAGE under reducing conditions and transferred to nitrocellulose membranes (Bio-Rad Laboratories). After blocking with 5% bovine serum albumin (BSA) in Tris-buffered saline (TBS) supplemented with 0.1% Tween-20, membranes were incubated overnight at 4°C with the following antibodies: rabbit anti-ACE2 (1:1000; abcam, ab272500), rabbit anti-phospho AMPKα Thr172 (1:1000; Cell signaling, 2531), and rabbit anti-AMPKα (1:1000; Cell signaling, 2532). Mouse anti-GAPDH (1:5000; Origene Technologies, TA802519) was used as sample-loading control. The signals were visualised on an Odyssey^®^FC Imaging System (LiCor) by infrared (IR) fluorescence using a secondary goat anti-rabbit IRDye 680LT antibody (1:1000; LiCor, FE3680210) and a goat anti-mouse IRDye 800CW (1:1000; LiCor, FE30926210). Bands were quantified through densitometry using the Image Studio Lite 5.0 (LiCor) software.

### Statistical Analysis

For studies in human subjects, data were expressed as mean ± standard deviation (SD) or as number of patients (%). Comparisons of binary characteristics in positive *vs* negative participants were performed using the chi-squared test, while age and continuous levels were compared with unpaired t-test. All analyses were carried out using SAS (Version 9.4). All *p-value*s were 2-sided.

For *in vitro* studies, all experiments were performed in at least three distinct biological samples (15 replicates for each sample). Data are presented as the mean ± standard error of the mean (SEM). Data analysis was performed using Prism Software (GraphPad Software Inc). Comparisons were made using unpaired t-test or ANOVA with Tukey *post hoc* test, as appropriate. Normality assumption was verified with the Shapiro-Wilk test. Statistical significance was defined as *p-value*<0.05.

## Data Availability Statement

The original contributions presented in the study are included in the article/**Supplementary Material**. Further inquiries can be directed to the corresponding author.

## Ethics Statement

The studies involving human participants were reviewed and approved by Azienda Sanitaria Locale Bergamo, Italy. The patients/participants provided their written informed consent to participate in this study.

## Author Contributions

Conceptualization: LP and MM. Investigation, data curation, and formal analysis: LP, MM, MG, APez, SG, and BI. Clinical data analysis: APer and PR. Evaluation of plasmatic complement components: RD. Writing of the original draft: LP, MM, MG, and BI. Supervision and final approval: AB and GR. All authors contributed to the article and approved the submitted version.

## Funding

The present study was supported by generous contributions from: Associazione Calcio Monza, Monza; Barabino Immobiliare, Milano; Brembomatic Pedrali, Pontirolo Nuovo, Bergamo; Fondazione Aiutiamoli a vivere, Ranica, Bergamo; Fondazione UBI Banca Popolare di Bergamo Onlus, Bergamo; GF-ELTI, Sovere, Bergamo; Mrs Mariella Guzzoni, Bergamo; Mrs Hazzazi Malika El, Milano; Mr Alberto Paccanelli, Bergamo; SMILAB Srl, San Pellegrino Terme, Bergamo; Mrs Paola Suardi, Bergamo. The funders had no role in study design, data collection or analysis, decision to publish, or preparation of the manuscript.

## Conflict of Interest

The authors declare that the research was conducted in the absence of any commercial or financial relationships that could be construed as a potential conflict of interest.

## Publisher’s Note

All claims expressed in this article are solely those of the authors and do not necessarily represent those of their affiliated organizations, or those of the publisher, the editors and the reviewers. Any product that may be evaluated in this article, or claim that may be made by its manufacturer, is not guaranteed or endorsed by the publisher.
