# Supplementary material for: SARS-CoV-2 Spike Protein 1 Activates Microvascular Endothelial Cells and Complement System Leading to Platelet Aggregation
Source: Front Immunol. 2022 Mar 7;13:827146. doi: 10.3389/fimmu.2022.827146 (PMC8936079; doi:10.3389/fimmu.2022.827146)
Supplement: Supplementary file 1 [file DataSheet_1.pdf]

A

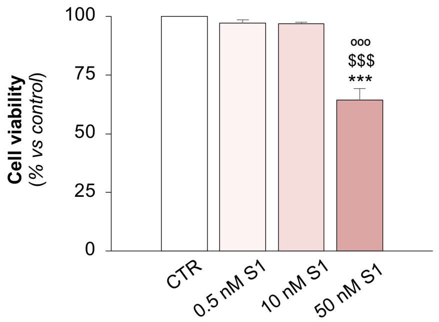

B

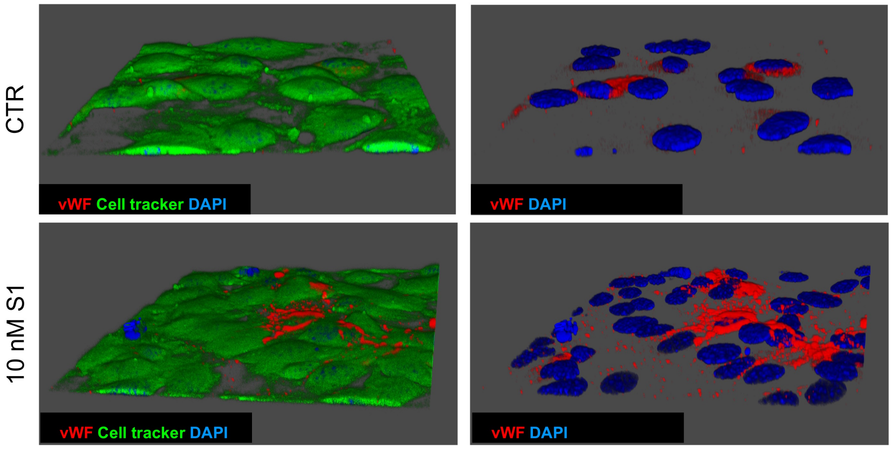

Supplementary Figure 1. Effect of S1 on cell viability and vWF. (**A**) Quantification of cell viability by crystal violet assay in HMEC-1 exposed for 24h to medium alone (CTR), 0.5 nM S1, 10 nM S1, or 50 nM S1. (**B**) Representative 3D reconstruction of z-stack slides of vWF staining (red) in HMEC-1 exposed to medium alone (CTR) or 10 nM S1. By staining endothelial cells with cell tracker (green), vWF is detectable in the cell cytoplasm of resting HMEC-1, while S1-activated endothelial cells exhibit a remarkable vWF staining on their luminal surface. Nuclei are counterstained with DAPI (blue). All experiments were repeated at least 3 times. Data represent mean ± SEM and were analysed with Tukey's multiple comparison test. ****p-value*<0.001 vs CTR; $^{\$\$\$}$*p-value*<0.001 *vs* 0.5 nM S1; ^{ooo}*p-value*<0.001 *vs* 10 nM S1.

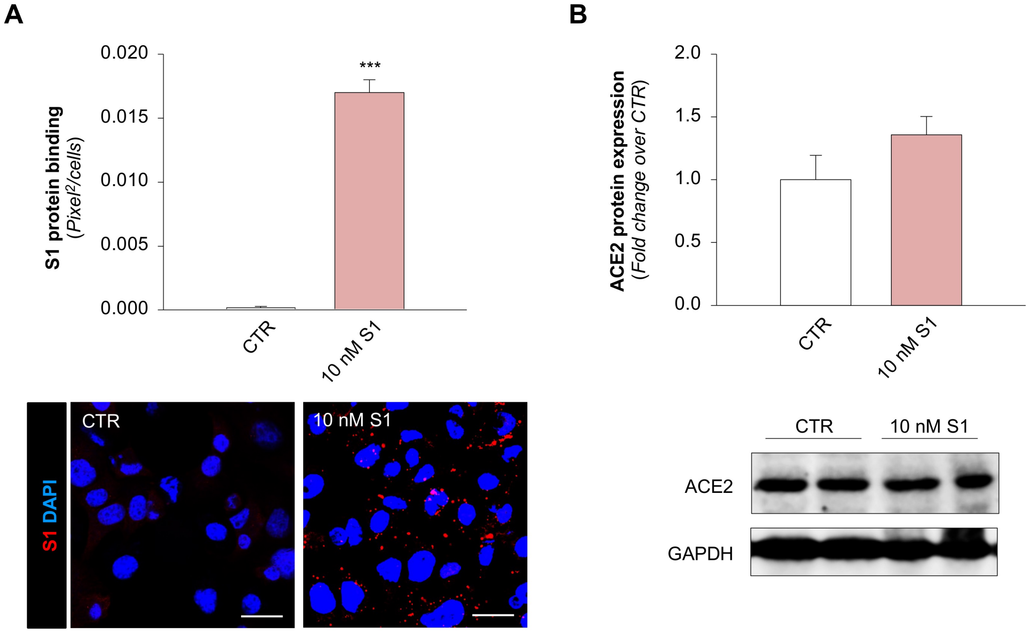

Supplementary Figure 2. Effect of S1 on VERO cells. (**A**) Quantification and representative images of binding of the S1 protein (red) on the apical surface of Vero cells exposed for 24h to medium alone (CTR) or 10 nM S1. Nuclei are counterstained with DAPI (blue). (**B**) Quantification and representative Western Blot of ACE2 protein expression in Vero cells exposed for 24h to medium alone (CTR) or 10 nM S1. GAPDH was used as a sample loading control. All experiments were repeated at least 3 times. Data represent mean ± SEM and were analysed with unpaired t-test. ****p-value*<0.001 *vs* CTR.

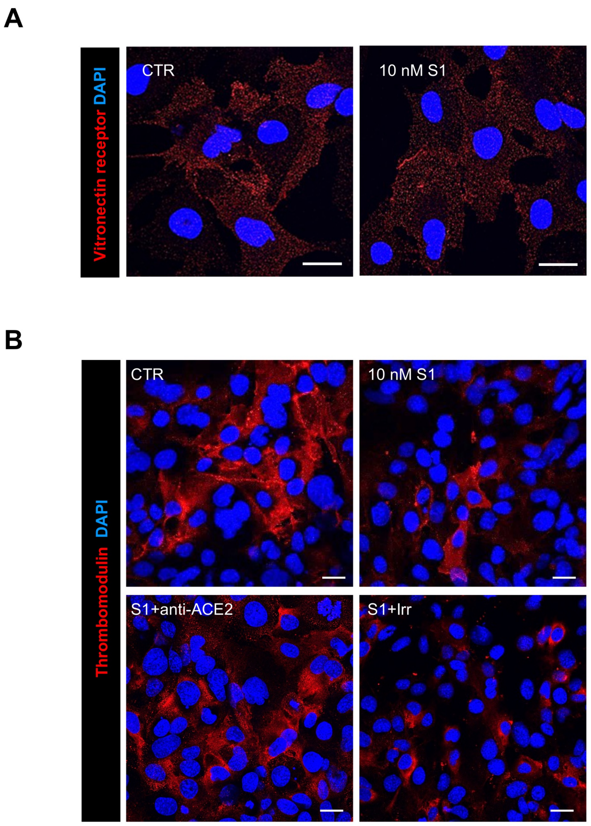

Supplementary Figure 3. Effect of S1 on vitronectin receptor and thrombomodulin expression. (**A**) Representative images of vitronectin receptor expression (red) on HMEC-1 incubated with medium alone (CTR) or S1 (10 nM). (**B**) Representative images of receptor thrombomodulin expression (red) on HMEC-1 incubated with medium alone (CTR) or S1 (10 nM) in the presence of anti-ACE2 Ab (2 μg/ml) or Irr Ab (2 μg/ml). Slides were counterstained with DAPI (blue). Scale bar 20 μm. All experiments were repeated at least 3 times.

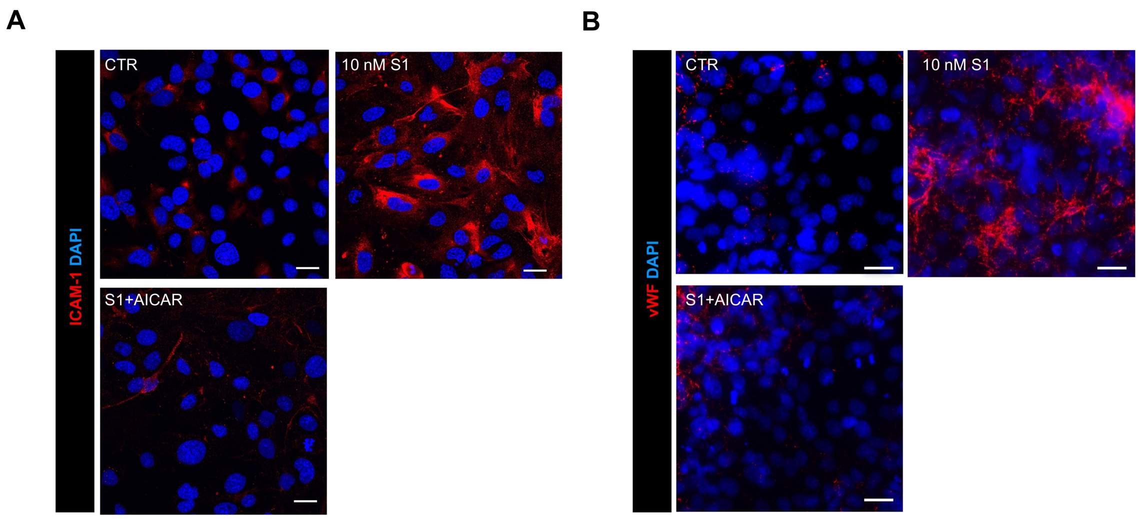

Supplementary Figure 4. Effect of S1 on ICAM-1 expression and vWF deposition. (**A,B**) Representative images of ICAM-1 expression (**A**, red) and vWF deposition (**B**, red) on HMEC-1 incubated with medium alone (CTR) or S1 (10 nM) in the presence or absence of AICAR (2 mM). Slides were counterstained with DAPI (blue). Scale bar 20 μm for panel a and 50 μm for panel b. All experiments were repeated at least 3 times.

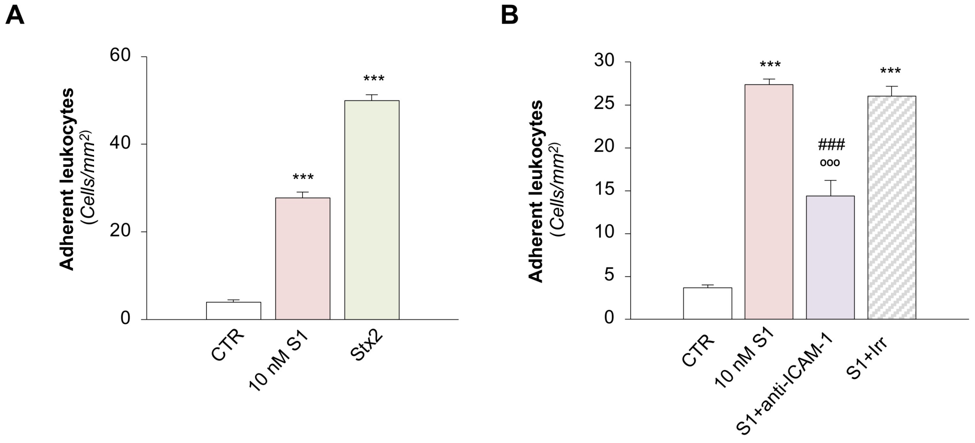

Supplementary Figure 5. Effect of S1 on leukocytes adhesion. (**A**) Quantification of leukocyte adhesion to HMEC-1 exposed for 24h to medium alone (CTR), S1 (10 nM) or Shiga Toxin 2 (Stx2, 50 pM) used as positive control. The number of leukocytes that adhered on HMEC-1 under flow conditions (1.5 dynes/cm^2) was estimated as Cells/mm^2. (**B**) Leukocyte adhesion to HMEC-1 exposed to medium alone (CTR) or S1 (10 nM) in the presence of anti-ICAM-1 functional blocking antibody (anti-ICAM-1, 10 μg/ml) or an irrelevant antibody (Irr, 10 μg/ml). All experiments were repeated at least 3 times. Data represent mean ± SEM and were analysed with Tukey's multiple comparison test. ****p-value*<0.001 *vs* CTR; °°°*p-value*<0.001 *vs* 10 nM S1; ###*p-value*<0.001 *vs* S1+Irr.

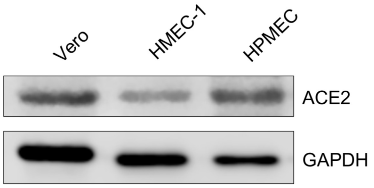

Supplementary Figure 6. Basal expression of ACE2 in different cell types. Representative Western Blot of constitutive ACE2 protein expression in Vero cells, HMEC-1, and HPMEC. GAPDH was used as a sample loading control.

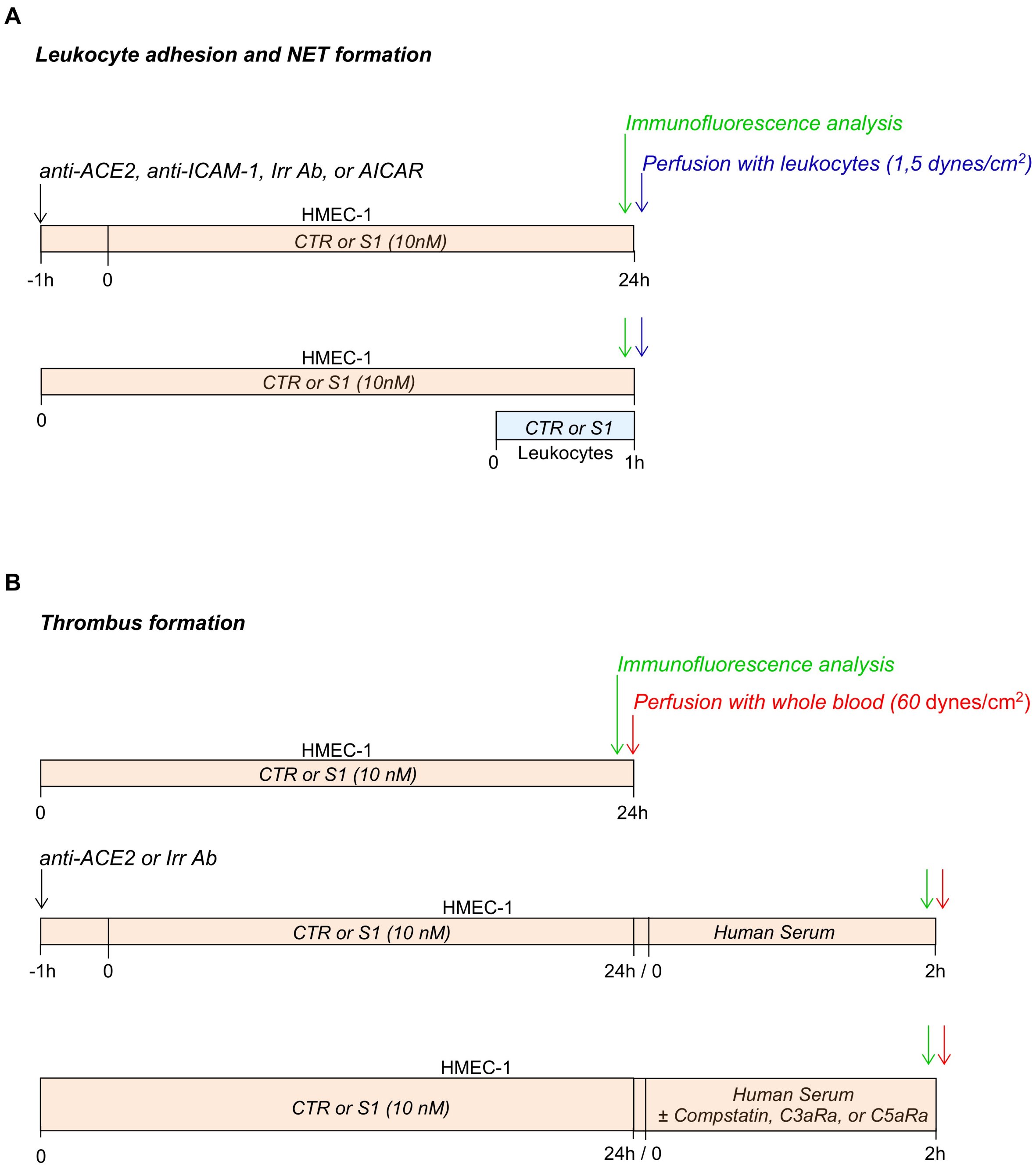

Supplementary Figure 7. Schematic representation of the experimental design. (**A,B**) Schematic representation of the experimental designs for all the experiments included in the study. Control, CTR; irrelevant, Irr; antibody, Ab; Angiotensin converting enzyme 2, ACE2; C3aR antagonist, C3aRa; C5aR antagonist, C5aRa.
